# Associating the risk of three urinary cancers with obesity and overweight: an overview with evidence mapping of systematic reviews

**DOI:** 10.1186/s13643-021-01606-8

**Published:** 2021-02-17

**Authors:** Jiyuan Shi, Liang Zhao, Ya Gao, Mingming Niu, Meili Yan, Yamin Chen, Ziwei Song, Xueni Ma, Peng Wang, Jinhui Tian

**Affiliations:** 1 Evidence-Based Nursing Centre, School of Nursing, Lanzhou University, Lanzhou City, 730000 China; 2 Evidence-Based Medicine Centre, School of Basic Medical Sciences, Lanzhou University, Lanzhou City, 730000 China; 3 School of Nursing and Health, Zhengzhou University, Zhengzhou city, 450001 China; 4 The Second Clinical Medical College of Lanzhou University, Lanzhou University, Lanzhou City, 730000 China

**Keywords:** Cancer, Obesity, Overweight, Meta-analysis

## Abstract

**Background:**

The relationship between cancer with overweight and obesity has been extensively reported. However, the association between urinary cancers with these risk factors remains unclear, with existing reports showing conflicting findings. The current review, therefore, sought to clarify the latter association by assessing the methodological and reporting quality of existing systematic reviews on the subject.

**Methods:**

We first screened PubMed, EMBASE, and Cochrane Library databases for relevant literature and subjected the resulting articles to meta-analysis. We adopted the AMSTAR-2 and PRISMA checklists for assessing methodological and reporting quality, respectively, then performed meta-analyses to determine the relationship between incidence and mortality of three types of urinary cancers with obesity and overweight. Indirect comparisons were also done across subgroups.

**Results:**

All systematic reviews (SRs) were of critically low methodological quality. Seventeen SRs had minimal reporting flaws, and 11 SRs had minor reporting flaws. We found an association between obesity with an incidence of kidney (RR = 1.68, 95% CI 1.47–1.92), bladder (RR = 1.1, 95% CI 1.07–1.13), and prostate (RR = 1.02, 95% CI 0.91, 1.13) cancers. Similarly, overweight was associated with the incidence of the three types of cancer, recording RR values of 1.37 (95% CI 1.26–1.48), 1.07 (95% CI 1.03–1.1), and 1 (95% CI 0.93, 1.07) for kidney, bladder, and prostate cancers, respectively. With regard to the dose analysis, the RR of BMI (per 5 kg/m^2^ increase) was associated with kidney (RR = 1.24, 95% CI 1.2–1.28), bladder (RR = 1.03, 95% CI 1.02–1.05), and prostate (RR = 1.02, 95% CI 1.01, 1.03) cancers.

**Conclusions:**

This comprehensive quantitative analysis provides an affirmation that overweight and obesity are strong risk factors for kidney cancer, owing to a strong association between them. Conversely, a weak association between overweight and obesity with bladder and prostate cancers confirms their status as mild risk factors for the 2 types of cancer. But due to the low quality of included SRs, the results need to be interpreted with caution.

**Systematic review registration:**

PROSPERO CRD42019119459

**Supplementary Information:**

The online version contains supplementary material available at 10.1186/s13643-021-01606-8.

## Mini-abstract

This umbrella review assessed the methodological and reporting quality of systematic reviews evaluating the relationship between three types of urinary cancer with obesity and overweight.

## Background

Cancer is the second most deadly disease affecting human health worldwide [1]. According to the Global Cancer Statistics of 2018, published by the World Health Organization/International Agency for Research on Cancer [2], prostate cancer represents the second most common type of cancer and the fifth leading cause of cancer-related deaths in men. For example, this type caused an estimated 1.3 million new cases and 359,000 deaths in 2018 alone, whereas bladder cancer accounted for 549,000 new cases and 20 million deaths worldwide. The incidence of bladder cancer (9.6/100,000) as well as mortality rate (3.2/100,000) is about four times (2.4/100,000 and 0.87/100,000) that of females. On the other hand, kidney cancer caused > 400,000 new cases and 170,000 deaths in the same year [2]. These figures underscore the incidence and impact caused by the three types of cancers across the world [2, 3], although the underlying mechanism of their development remains unclear owing to limited evidence. Previous studies have suggested that cumulative effects of cigarette smoking, alcohol consumption, obesity, and genetic susceptibility may be risk factors for urinary cancer [3, 4]. Accurate understanding of these risk factors is critical for the development of effective approaches for cancer prevention and treatment.

Overweight and obesity are defined as excess body weight that causes many chronic diseases and increases the risk of death. The number of overweight and obese adults had risen to 2.1 billion in 2013, with direct costs resulting from obesity estimated to account for 0.7–2.8% of a country’s total healthcare expenditures [5, 6]. In the USA, Wang et al. [7] predicted a $48–66 billion increase per year in combined medical costs from common obesity-related diseases by 2030. Policymakers in the public health sector rely on high-quality evidence, generated by meta-analyses and systematic reviews (SRs), to formulate policies for the prevention and management of cancer. However, despite numerous SRs describing the relationship between cancer and BMI, overweight, and obesity, over the past several decades, the quality of them has not been evaluated, which is an essential step before recommendations were presented and applied confidently; on the other hand, complexing findings regarding the association of these risks factors with urinary cancers pose a challenge to accurate understanding of their epidemiology as well as the development of management approaches [8–12]. An overview of systematic reviews (OoSRs) is a study designed to synthesize multiple evidence from existing systematic reviews on a specific domain, which have been developed to address the growing problem of information overload, providing a way to filter large bodies of complex evidence in order to inform healthcare decision-making [9–12].

In the current study, we sought to generate more comprehensive and robust evidence of the relationship between the aforementioned urinary cancers and obesity and overweight using a meta-analysis of published literature.

## Material and methods

### Protocol registration

This overview was registered by the PROSPERO (International Prospective Register of Systematic Reviews), number CRD42019119459. This overview was conducted following the Preferred Reporting Items for OoSRs (PRIO-harms) checklist.

### Search strategy and selection criteria

Two authors independently searched the PubMed, Cochrane Library, and EMBASE databases in March 2019. The search was limited to articles written in English and used the following search terms: BMI, obesity, cancer, carcinoma, neoplasm, meta-analysis, and SRs. A detailed description of the search strategy is presented in the supplementary material. In addition, we conducted supplementary retrieval of all included references and updated the search in November 2020 to enable a comprehensive search.

### Eligibility and inclusion/exclusion criteria

Studies were included if (1) SRs or meta-analysis associated obesity and overweight with incidence and mortality of aforementioned urinary cancer, (2) SRs or meta-analysis associated increase in BMI with incidence and mortality of aforementioned urinary cancer, (3) articles were published in English, and (4) latest article was included when SRs or meta-analysis had been updated. Studies were excluded if (1) they were only abstracts and/or letters; (2) SRs and meta-analysis examined the association between BMI increase and prognosis, survival, or recurrence of urinary cancers; (3) protocols of SRs and meta-analysis or methodological articles; and (4) SRs without meta-analysis.

### Study selection and data retrieval

The retrieved articles were first imported into the EndNote X7 software, then titles and abstracts independently selected by two reviewers. The reviewers thereafter retrieved full texts of potentially eligible studies and independently subjected them to the aforementioned criteria (J.Y.S and X.N.M.). Any disagreement was discussed with a third reviewer. The two reviewers independently extracted the following characteristic from each study: first author’s name, year of publication, funding, number of reference test, name of the database, country of the first author, the epidemiological study design (case-control or cohort), number of cases, features of the urinary cancers, summary effects between BMI and cancer risk (at 95% CI), and the number of included studies.

### Methodological and reporting quality assessment

The quality assessment of included SRs was independently performed by two review authors (Y.G. and J.Y.S.) according to the predefined criteria. Disagreements regarding by-item and overall rating of quality were resolved by consensus or third-party adjudication (P.W. or J.H.T.). The methodological quality was assessed using the assessment of multiple systematic reviews-2 (AMSTAR-2) tool, which is a reliable methodological quality tool applicable to SRs of randomized and/or non-randomized studies with good agreement, construct validity, and feasibility. The AMSTAR-2 contains 16 items, among which seven are critical domains. Each item was responded to “yes” (item/question fully addressed), “no” (item/question not addressed), or “partial” (item/question not fully addressed). The overall confidence of the quality of each SR was classified as high, moderate, low, or critically low according to the critical and non-critical domains [13, 14].

To assess the reporting quality of included SRs and meta-analyses, we used the PRISMA checklist, which comprises 27 items. To show the degree of compliance, total PRISMA scores were calculated by summing 1 point for “yes” (total confirmed), 0.5 points for “partial” (partial confirmed) and “cannot answer” (limited information), and 0 points for “no” (non-compliance) [15]. The SR and meta-analysis were regarded as major flaws if PRISMA scores were below 15 points, minor flaws if they recorded 15.0–21.0 points, and minimal flaws if > 21.0 points were recorded [14, 16]. Quality assessment of the included SRs and meta-analyses was independently performed by two authors (J.Y.S. and Y.G.), and any disagreements between them were discussed with a third author (J.H.T.).

### Statistical analysis

We created a bubble plot with Microsoft Excel 2016 (Microsoft Corp., Redmond, WA, www.microsoft.com) to present the compliance of AMSTAR-2 and PRISMA of the included SRs. The evidence map displayed information in four dimensions: (a) the *X*-axis displayed different items of AMSTAR-2 or PRISMA; (b) the *Y*-axis represented the number of each item of AMSTAR-2 or PRISMA defined as “yes,” “partial,” and “no”; (c) the bubble size represented the compliance of each item of AMSTAR-2 or PRISMA defined as “yes,” “partial,” and “no”; (d) the bubble color indicated the items of AMSTAR-2 or PRISMA defined as “yes,” “partial,” and “no.”

The pairwise meta-analysis was performed with the data of pooled RRs (at 95% CI) from included SRs and meta-analysis (HRs and ORs equivalent to RR) records using the fixed-effects model or random-effects model [17]. A condition was considered normal if a BMI of 18.5–24.9 kg/m^2^ was recorded, overweight for 25–29.9 kg/m^2^, and obesity for BMI ≥ 30 kg/m^2^. We also analyzed each 1 kg/m^2^ and 5 kg/m^2^ increase in BMI according to previous protocols [18, 19]. In addition, we assessed the heterogeneity between studies using the *I*^2^ statistic [20]. Specifically, we adopted the fixed-effect model when the *I*^2^ value was less than 50%, and the random-effect model for an *I*^2^ value was greater than 50% [21]. We carried out subgroup analyses according to the sex, location, history of smoking, age, family history of cancer, duration of follow up, different types of study (e.g., cohort study or case-control study), physical activity, methods of BMI measured, history of hypertension, history of alcohol drinking if data were sufficient, and if the definition of the subgroup is different, only the high methodological quality results were included. Furthermore, we analyzed the indirect comparisons of the outcomes across the meta-analyses. Statistical analyses were performed using the STATA software (version 12.0, College Station, TX, USA), with values that had *P* ≤ 0.05 considered statistically significant.

## Results

### Search results

**Fig. 1:**
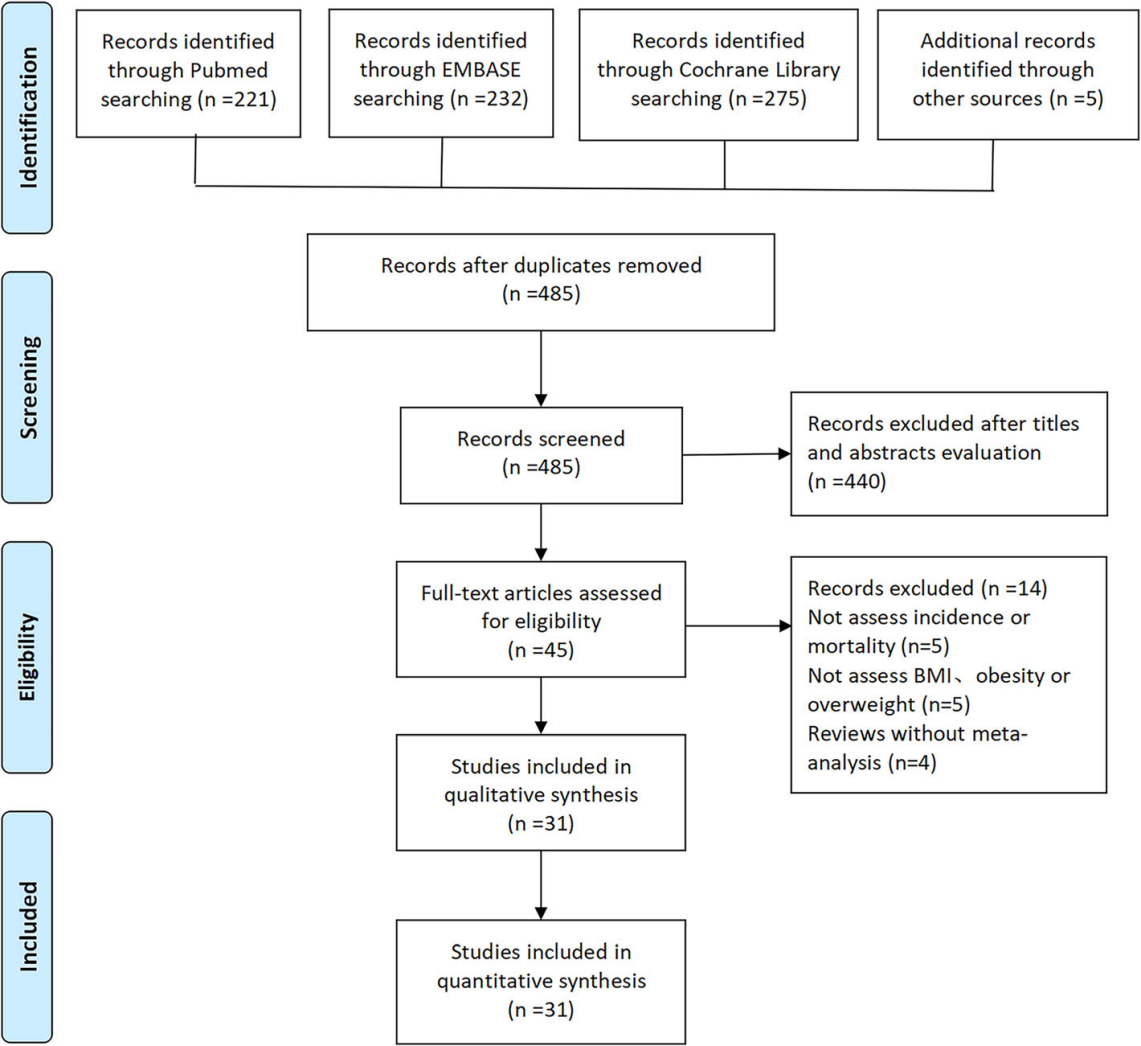
Flow diagram of the literature search

Our search resulted in a total of 733 articles, with 221, 232, and 275 identified from PubMed, EMBASE, and Cochrane Library, respectively. Additional five were retrieved from reference lists or other sources. A screening flow chart of the studies is presented in Fig. 1. After retrieval, a total of 248 duplicates were removed. A review of full-text articles resulted in the exclusion of 14 studies, with 31 SRs and meta-analyses meeting our criteria. Of these, 7 described mortality, 22 reported incidence, and 2 were studies combining mortality and incidence. A detailed description of these characteristics is outlined in Table 1.

**Table 1:** Study characteristic

Study	Year	Compliance of AMSTAR/overall confidence	PRISMA scores	Cancer	Study design	Effect size	No. of studies
Qin [22]	2013	9/critically low	21	Bladder cancer	Cohort	RR	11
Sun [23]	2015	9/critically low	21	Bladder cancer	Cohort	RR	15
Zhao [24]	2017	13/critically low	23	Bladder cancer	Cohort	RR	14
Bagheri [25]	2016	7/critically low	17.5	Kidney cancer	Cohort	HR	8
Bergström [26]	2001	4/critically low	15.5	Kidney cancer	Cohort, case-control	RR	29
Ildaphonse [27]	2009	3/critically low	12.5	Kidney cancer	Cohort	OR, RR	27
Mathew [28]	2009	4/critically low	13	Kidney cancer	Cohort	OR, RR	28
Wang [29]	2014	7/critically low	23	Kidney cancer	Cohort	RR	21
Zhang [30]	2018	10/critically low	21.5	Kidney cancer	Cohort	HR	19
Chen [31]	2016	9/critically low	22.5	Prostate cancer	Cohort, case-control	RR	9
Discacciati [32]	2012	9/critically low	19.5	Prostate cancer	Cohort	RR	12
Jiang [33]	2017	12/critically low	19.5	Prostate cancer	Cohort	RR	9
MacInnis [34]	2006	6/critically low	24	Prostate cancer	Cohort	RR	56
Xie [35]	2017	10/critically low	20.5	Prostate cancer	Cohort	RR	21
Zhang [36]	2015	9/critically low	18.5	Prostate cancer	Cohort, case-control	RR	17
Zhong [37]	2016	8/critically low	24.5	Prostate cancer	Cohort, case-control	RR	24
Guh [38]	2009	8/critically low	18.5	Prostate cancer Kidney cancer	Cohort	RR	13
Fang [39]	2018	11/critically low	23	Prostate cancer Kidney cancer Bladder cancer	Cohort	RR	87
Al-Zalabani [40]	2016	10/critically low	25.5	Bladder cancer	Cohort	RR	26
Wang [41]	2016	9/critically low	24.5	Prostate cancer Kidney cancer	Cohort	RR	59
Robinson [42]	2008	7/critically low	21.5	Prostate cancer	Cohort, case-control	RR	16
Renehan [43]	2008	12/critically low	25	Prostate cancer Kidney cancer	Cohort, case-control	RR	44
Bergstom [44]	2001	4/critically low	14.5	Prostate cancer Kidney cancer	Cohort, case-control	RR	17
Cao [45]	2011	9/critically low	19	Prostate cancer	Cohort	RR	8
Xue [46]	2017	11/critically low	23.5	Kidney cancer Bladder cancer	Cohort	RR	24
Wang [47]	2008	8/critically low	24	Kidney cancer	Cohort, case-control	RR	44
Dobbins [48]	2013	7/critically low	20.5	Prostate cancer	Cohort, case-control	RR	5
Liu [49]	2018	9/critically low	23.5	Kidney cancer	Cohort	RR	24
Hidayat [50]	2018	9/critically low	22	Kidney cancer Prostate cancer	Cohort, case-control	RR	18
Harrison et al. [51]	2020	12/critically low	25	Prostate cancer	Cohort, case-control	OR	21
Berger et al. [52]	2019	12/critically low	26	Prostate cancer	Cohort	RR	12

### Study characteristics

Characteristics of included SRs and meta-analyses included in the current study are outlined in Table 1 [22–52]. Among the 31 records that met our inclusion criteria, 4 SRs reported bladder cancer cases [22–24, 40], 9 SRs and meta-analyses focused on kidney cancer [25–30, 47–49], 11 SRs and meta-analyses described prostate cancer [31–37, 42, 45], and 7 meta-analyses studied more than one type of urinary cancers [38, 39, 41, 43, 44, 46, 50]. The included SRs and meta-analyses were published between 2001 and 2018, with only one record retrieved from Chinese databases [22]. One record had co-first authors [24], whereas the median number of authors was 4.5 and ranged from 2 to 10. In addition, five articles did not report sources of funding [26–30], five others provided full search strategy [29, 39, 40, 43, 46], and only one was registered with PROSPERO [40].

### Quality assessment

**Fig. 2:**
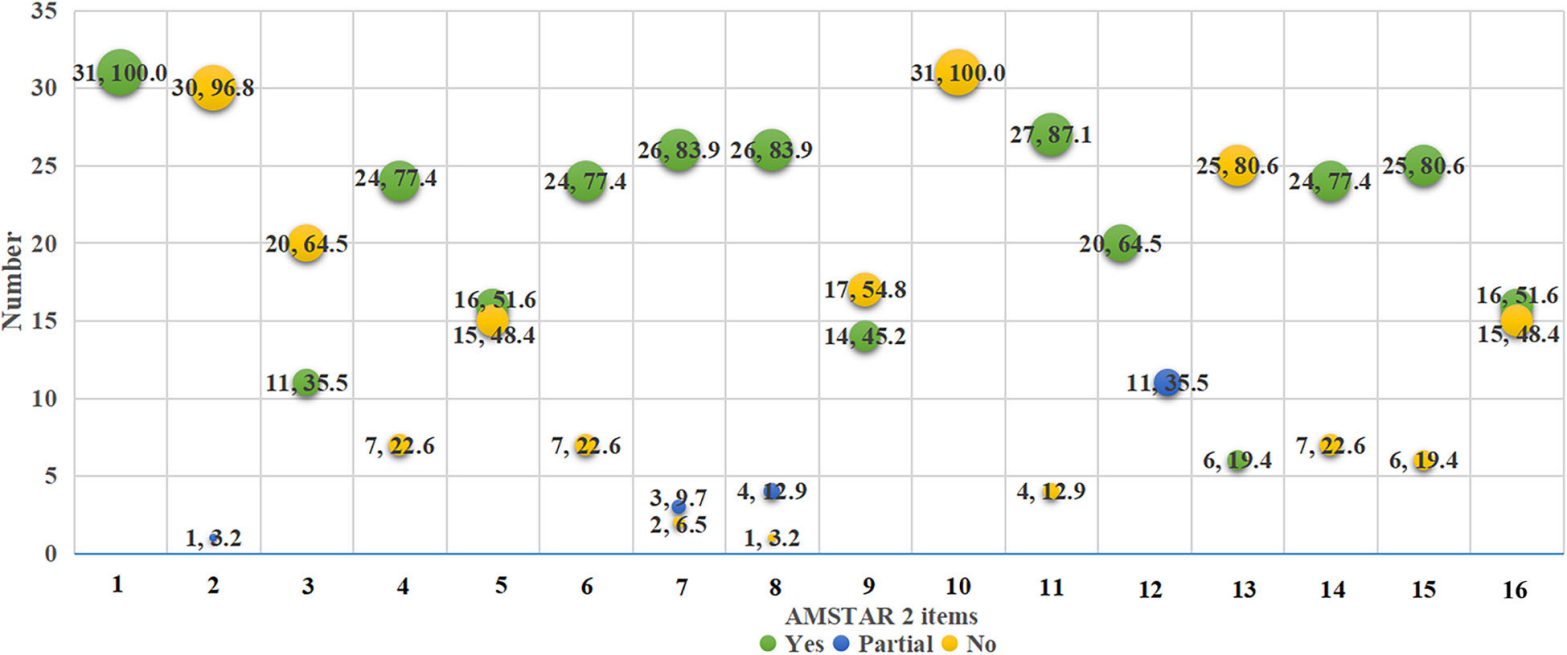
Datasets by AMSTAR-2 item score. The full compliance rate of each AMSTAR-2 item

**Fig. 3:**
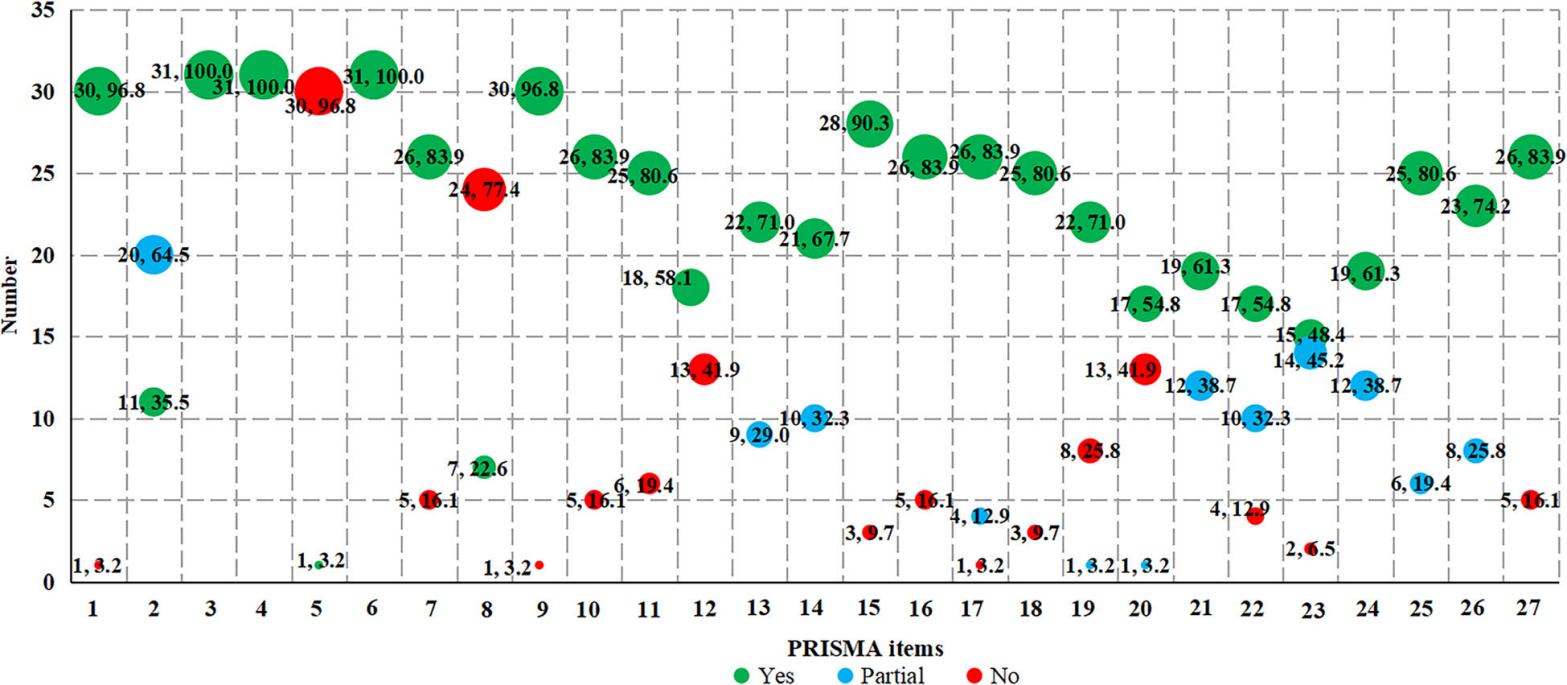
Datasets by PRISMA item score. The full compliance rate of each PRISMA item

Thirty-one SRs were available for quality assessment. Considering the overall methodological quality, all the SRs were rated as critically low quality. Specifically, of the sixteen individual items, one was fully reported (research questions and inclusion criteria for the review include the components of PICO). However, none of the SRs explicitly stated that the review methods were established before the conduct of the review and justified significant deviations from the protocol, and report on the sources of funding for the studies included in the review (Fig. 2). In general, 72.41% of the articles were of moderate quality (Table 1). The total of PRISMA points across the included studies was 21.5 (12.5–26) (Fig. 3). Particularly, four items were fully reported, whereas only one [40] provided the protocol’s registration number. In addition, three meta-analyses [27, 28, 44] had low reporting quality (PRISMA score < 15.0), 11 [22, 23, 25, 26, 32, 33, 35, 36, 38, 45, 48] were moderate (15.5–21.0), and 17 articles [24, 29–31, 34, 37, 39–43, 46, 47, 49–52] were of high quality (21.5–26.0) (Table 1).

#### Bladder cancer

A total of four studies evaluated the relationship between the incidence of bladder cancer and obesity [23, 24, 40, 46], with pooled estimates of 1.1 (95% CI 1.07–1.13) (Table 2). A subgroup analysis revealed a stronger relationship between obesity and the risk of prostate cancer in North America [22–24] compared to Europe (*P* < 0.05) [22–24]. In addition, obese people who drink alcohol were reported to be more likely to develop bladder cancer compared to their non-drinking counterparts (*P* < 0.05) [23]. Age [22, 23], smoking [22–24], gender [22, 23, 46], and duration of follow-up (10 years) [23, 24] were not significant confounding factors for the disease (Table 3).

**Table 2:** Meta-analysis on the relationship between obesity, overweight, and BMI and incidence of urinary cancer

Cancer type	Overweight		Obesity		Per 5 kg/m^2^		Per 1 kg/m^2^	
	RR (95% CI)	Combined RR (95% CI)	RR (95% CI)	Combined RR (95% CI)	RR (95% CI)	Combined RR (95% CI)	RR (95% CI)	Combined RR (95% CI)
Bladder	Sun et al. [23] 1.07 (1.01, 1.14) Zhao et al. [24] 1.03 (0.95, 1.11) Al-Zalabani et al. 1.07 (0.99, 1.16) Xue et al. [40] 1.09 (1.01, 1.17)	1.07 (1.03, 1.1)	Qin [22] 1.1 (1.06, 1.16) Zhao et al. [24] 1.1 (1.03, 1.17) Sun at al [23]. 1.1 (1.06, 1.14) Al-Zalabani et al. [40] 1.1 (1.03, 1.18) Xue et al. [46] 1.48 (0.89, 2.45)	1.1 (1.07, 1.13)	Zhao et al. [24] 1.03 (1.01, 1.06) Fang et al. [39] 1.03 (1, 1.07)	1.03 (1, 1.06)	Bergström at al [44]. 1.03 (1, 1.06)	1.03 (1, 1.06)
Kidney	Wang et al. [29] 1.28 (1.24, 1.33) Daphne et al. [38] 1.55 (1.47, 1.63) Xue et al. [46] 1.34 (1.11, 1.62) Wang et al. [29] 1.31 (1.23, 1.4) Liu et al. [49] 1.35 (1.27, 1.43)	1.37 (1.26, 1.48)	Wang et al. [29] 1.77 (1.68, 1.87) Daphne et al. [38] 2.2 (1.53, 3.16) Xue et al. [46] 1.76 (1.47, 2.1), Wang et al. [47] 1.71 (1.53, 1.93) Dobbins et al. [48] 1.67 (1.55, 1.79) Liu et al. [49] 1.76 (1.61, 1.91)	1.68 (1.47, 1.92)	Wang et al. [41] 1.25 (1.17, 1.33) Fang et al. [39] 1.2 (1.16, 1.25) Renehan et al. [43] 1.29 (1.2, 1.39) Daphne et al. [38] 1.22 (1.16, 1.28)	1.24 (1.2, 1.28)	Bergström et al. [26] 1.07 (1.04, 1.09) Wang et al. [29] 1.04 (1.03, 1.05) Bergström et al. [44] 1.06 (1.05, 1.34)	1.05 (1.03, 1.08)
Prostate	Daphne et al. [38] 1 (0.95, 1.06) Harrison et al. [51] 0.99 (0.91, 1.08)	1 (0.93, 1.07)	Daphne et al. [38] 1.14 (1, 1.31) Harrison et al. [51] 0.90 (0.81, 1.01)	1.02 (0.91, 1.13)	Discacciati et al. [32] 1.03 (0.99, 1.06) MacInnis et al. [34] 1.05 (1.01, 1.08) Xie et al. [35] 1.07 (1.03, 1.12) Fang et al. [39] 1.01 (0.98, 1.03) Wang et al. [41] 1.03 (1.01, 1.05) Robinson et al. [42] 1.08 (0.97, 1.9) Dobbins et al. [48] 1.06 (0.99, 1.14) Harrison et al. [51]e 0.99 (0.96, 1.02) Berger et al. [52] 1.02 (0.94, 1.11)	1.02 (1.01, 1.03)	Bergström at al [44].	1.01 (1, 1.02)

**Table 3:** Subgroup of the relationship between obesity, overweight, and BMI and incidence of bladder cancer

Stratification criteria		Overweight, RR (95% CI)	Obesity, RR (95% CI)	Per 5 kg/m^2^, RR (95% CI)
Sex	Male	1.09 (0.99, 1.2)	1.1 (1.05, 1.16)	1.05 (1, 1.1)
	Female	1.06 (0.99, 1.14)	1.08 (1.02, 1.14)	1.02 (0.96, 1.09)
	*P* value	0.647	0.185	0.474
Age	Age < 50	1.04 (0.97, 1.13)	1.08 (1.02, 1.14)	
	Age ≥ 50	1.11 (1.03, 1.19)	1.15 (1.1, 1.2)	
	*P* value	0.224	0.081	
Study location	Asia	1.04 (0.99, 1.09)	1.02 (0.97, 1.08)	
	North America	1.12 (1.03, 1.22)	1.13 (1.07, 1.19)	1.06 (1, 1.12)
	Europe	1.09 (0.98, 1.2)	1.06 (1.01, 1.12)	1.02 (0.98, 1.06)
	Asia versus North America (*P* value)	0.136	0.008	
	Asia versus Europe (*P* value)	0.412	0.312	
	North America versus Europe (*P* value)	0.687	0.091	0.274
Measure	BMI measured	1.1 (1, 1.2)	1.09 (1.05, 1.13)	
	BMI self-measured	1.12 (1.04, 1.19)	1.18 (1.1, 1.27)	
	*P* value	0.755	0.054	
Duration	Duration of follow-up < 10 years	1.07 (1.03, 1.11)	1.1 (1.05, 1.16)	
	duration of follow-up ≥ 10 years	1.07 (0.98, 1.17)	1.1 (1.04, 1.15)	
	*P* value	1.000	1.000	
Physical	Physical activity	1.11 (0.99, 1.24)	1.2 (1.08, 1.33)	
	No physical activity	1.03 (1, 1.07)	1.09 (1.04, 1.13)	
	*P* value	0.212	0.093	
History	Family history of cancer	1.15 (1.05, 1.26)	1.15 (1.09, 1.2)	
	No family history of cancer	1.06 (1.01, 1.11)	1.09 (1.06, 1.13)	
	*P* value	0.120	0.069	
Smoking	Smoking	1.07 (0.99, 1.16)	1.1 (1.04, 1.15)	
	No smoking	1.1 (1.05, 1.15)	1.11 (1.07, 1.15)	
	*P* value	0.553	0.774	
Alcohol	Alcohol	1.13 (1.04, 1.22)	1.17 (1.06, 1.3)	
	No alcohol	1.06 (0.99, 1.14)	1.09 (1.05, 1.13)	
	*P* value	0.443	0.002	

The relationship between the incidence of bladder cancer and overweight was described by four articles [23, 24, 40, 46], with pooled estimates of 1.07 (95% CI 1.03–1.1) (Table 2). A subgroup analysis revealed that gender [22–24], geographic location [23, 24], age [23, 24], duration of follow-up (10 years) [23, 24], family history of cancer [23, 24], physical activity [23, 24], and alcohol consumption [23] were not confounding factors in the relationship (Table 3). Furthermore, we observed a linear relationship between bladder cancer with BMI for each per 5 kg/m^2^ [24, 39] (RR = 1.03, 95% CI 1.02–1.05). The RR for every 1 kg/m^2^ BMI increment might also be related to bladder cancer (1.03, 1–1.06) [53].

#### Prostate cancer

Two studies evaluated the association between the incidence of prostate cancer with obesity and overweight [36, 38], resulting in pooled estimates of RR = 1.02 (95% CI 0.91, 1.13) and 1 (95% CI 0.93–1.07), respectively (Table 2). We also observed a linear relationship between the incidence of prostate cancer and BMI for every per 5 kg/m^2^ [32, 34, 35, 39, 41, 42, 48] (Table 2). In addition, the relationship between BMI increase and incidence of prostate cancer was stronger in Asia [42] compared to Europe [39, 41, 42] and North America (*P* < 0.05) [39, 41, 42]. Duration of follow-up (10 years) (*P* = 0.131) and BMI self-measure (*P* = 0.397) were not confounding factors for prostate cancer [41] (Table 4).

**Table 4:** Subgroup of the association between BMI and incidence of prostate cancer

Stratification criteria		Per 5 kg/m^2^, RR (95% CI)
Study location	Asia	1.23 (0.79, 1.92)
	North America	1.03 (1, 1.05)
	Europe	1.01 (0.97, 1.05)
	Asia versus North America (*P* value)	0.033
	Asia versus Europe (*P* value)	0.014
	North America versus Europe (*P* value)	0.409
Measure	BMI measured	1.04 (1.01, 1.07)
	BMI self-measured	1.02 (0.98, 1.05)
	*P* value	0.397
Duration	Duration of follow-up ≥ 10 years	1.01 (0.99, 1.04)
	Duration of follow-up < 10 years	1.04 (1.01, 1.07)
	*P* value	0.131

#### Kidney cancer

A total of 6 studies [29, 38, 46–49] described the relationship between the incidence of kidney cancer and obesity, resulting in a strong pooled estimate RR = 1.68 (95% CI 1.47–1.92) (Table 2). Obesity had a stronger association with the incidence of kidney cancer in women (RR = 2, 95% CI 1.91–2.08) than in men (RR = 1.65, 95% CI 1.56–1.74) [29, 43, 46, 48]. Similarly, a stronger association was observed between obesity and the incidence of kidney cancer in North America relative to Europe [29, 49]. In addition, two studies [29, 49] analyzed the alcohol consumption subgroup, with an indirect comparison showing a weaker association between the incidence of kidney cancer and obesity in the alcohol-drinking compared to no alcohol groups (*P* < 0.05). A separate analysis based on age showed a higher RR in the young (age < 50) compared to the older group [39, 49] (RR = 1.7, 95% CI 1.62–1.78) (*P* < 0.05) (Table 5).

**Table 5:** Subgroup of the association between obesity, overweight, and BMI and incidence of kidney cancer

Stratification criteria		Overweight, RR (95% CI)	Obesity, RR (95% CI)	Per 5 kg/m^2^, RR (95% CI)
Sex	Male	1.28 (1.16, 1.41)	1.65 (1.56, 1.74)	1.18 (1.13, 1.23)
	Female	1.51 (1.32, 1.72)	2 (1.91, 2.08)	1.27 (1.23, 1.32)
	*P* value	0.049	0.000	0.009
Age	Age < 50	1.43 (1.3, 1.57)	2.05 (1.84, 2.28)	
	Age ≥ 50	1.41 (1.37, 1.45)	1.7 (1.62, 1.78)	
	*P* value	0.779	0.020	
Study location	Asia	1.59 (1.41, 1.8)	2.06 (1.26, 3.37)	1.23 (0.79, 1.92)
	North America	1.41 (1.33, 1.46)	1.9 (1.77, 2.04)	1.19 (1.1, 1.28)
	Europe	1.22 (1.18, 1.27)	1.67 (1.58, 1.77)	1.19 (1.14, 1.25)
	Asia versus North America (*P* value)	0.072	0.750	0.031
	Asia versus Europe (*P* value)	0.000	0.406	0.013
	North America versus Europe (*P* value)	0.000	0.005	1.000
Measure	BMI measured	1.26 (1.21, 1.31)	1.69 (1.59, 1.8)	1.24 (1.14, 1.35)
	BMI self-measured	1.35 (1.28, 1.42)	1.86 (1.74, 1.99)	1.15 (1.11, 1.2)
	*P* value	0.755	0.400	0.113
Duration	Duration of follow-up ≥ 10 years	1.28 (1.23, 1.33)	1.78 (1.67, 1.89)	
	Duration of follow-up < 10 years	1.32 (1.19, 1.45)	1.76 (1.57, 1.97)	
	*P* value	0.570	0.864	
Physical	Physical activity	1.33 (1.24, 1.42)	1.75 (1.58, 1.94)	
	No physical activity	1.27 (1.21, 1.32)	1.78 (1.67, 1.9)	
	*P* value	0.261	0.780	
Hypertension	Hypertension	1.36 (1.25, 1.49)	1.93 (1.74, 2.16)	
	No hypertension	1.27 (1.22, 1.32)	1.72 (1.62, 1.83)	
	*P* value	0.163	0.069	
Alcohol	Alcohol	1.29 (1.21, 1.38)	1.62 (1.49, 1.75)	
	No alcohol	1.22 (1.16, 1.29)	1.82 (1.72, 1.92)	
	*P* value	0.685	0.019	
Smoking	Smoking	1.25 (1.21, 1.29)	1.1 (1.04, 1.15)	
	No smoking	1.1 (1.05, 1.15)	1.11 (1.07, 1.15)	
	*P* value	0.077	0.293	

On the other hand, five studies [29, 38, 46, 47, 49] associated the incidence of kidney cancer and overweight, resulting in a pooled RR of 1.37 (95% CI 1.26–1.48). Overweight had a stronger relationship with the incidence of kidney cancer in women (RR = 1.51, 95% CI 1.32–1.72) compared to men (RR = 1.28, 95% CI 1.16–1.41) (*P* = 0.049). Asia had a stronger association (RR = 1.59, 95% CI 1.41–1.8) than North America (RR = 1.41, 95% CI 1.33–1.46) and Europe [29, 49] (RR = 1.22, 95% CI 1.18–1.27). In addition, indirect comparisons revealed a significantly stronger positive association between obesity and incidence of cancer in the Asian group, compared to those from North America (RR = 1.13, 95% CI 0.99–1.29, *P* = 0.072) and Europe (RR = 1.3, 95% CI 1.15–1.48, *P* = 0). Smoking, hypertension, physical activity, BMI self-measures, duration of follow-up (10 years), and alcohol drinking [29, 49] were not confounding factors (Tables 2 and 5).

We observed a linear relationship incidence of kidney cancer and BMI for every per 5 kg/m^2^ [38, 39, 41, 43, 46] (RR = 1.24, 95% CI 1.2–1.28) (Table 5). In addition, increased BMI was strongly associated with the incidence of kidney cancer in women compared to men (*P* < 0.05) [41, 43]. With regard to the regions, the Asia group recorded a stronger positive association, between BMI increase and cancer, than North America and Europe (*P* < 0.05). RRs for every 1 kg/m^2^ in BMI increase [26, 29] (RR = 1.05, 95% CI 1.03–1.08) revealed a mild relationship.

### Association between BMI increase and mortality rates from urinary cancers

A summary of the effect of each 5 kg/m^2^ BMI increase on mortality rates resulting from urinary cancers is provided in Table 6. Specifically, mortality rates of bladder [39] (1.05, 95% CI 1–1.11), kidney [39] (1.21, 95% CI 1.14–1.29), and prostate [32, 33, 39, 42, 45] (1.15, 95% CI 1.11–1.2) cancers were all associated with BMI increase. Zhang et al. [30] reported a strong association between mortality rates from kidney cancer with obesity (RR = 1.71, 95% CI 1.27–2) and overweight (RR = 1.19, 95% CI 1.05–1.35), whereas Zhong et al. [37] observed that prostate cancer-related mortality was strongly associated with per 5 kg/m^2^ BMI increase (HR = 1.15, 95% CI 1.07–1.23). Conversely, each 5 kg/m^2^ BMI increment showed no effect on all-cause mortality in prostate cancer patients.

**Table 6:** Association between BMI and mortality of urinary cancers

Cancer type	Mortality type	Per 5 kg/m^2^	
		RR (95% CI)	Combined RR (95% CI)
Bladder	Mortality	Fang et al. [39] 1.05 (1–1.11)	1.05 (1–1.1)
Kidney	Mortality	Fang et al. [39] 1.21 (1.14–1.29)	1.21 (1.14–1.29)
Prostate	Mortality	Jiang et al. [33] 1.16 (1.1–1.23), Fang et al. [39] 1.11 (1.06–1.15), Cao et al. [45] 1.15 (1.06–1.25)	1.15 (1.11–1.2)
Prostate	Cause-specific mortality	Zhong et al. [37] 1.1 (0.99–1.22), Cao et al. [45] 1.2 (0.99–1.46)	1.12 (1.02–1.23)
Prostate	All-cause mortality	Zhong et al. [37] 1.05 (0.97–1.12)	1.05 (0.97–1.12)

## Discussion

Despite numerous studies analyzing cancer risk factors [2], no consensus has been reached regarding the relationship between urinary cancer and obesity and overweight. Consequently, no evidence-based decision-making can be done using existing information, necessitating further studies. In the current study, we identified 31 SR or meta-analysis articles describing the relationship between urinary cancers and obesity and overweight, then used the AMASAR-2 and PRISMA checklists to assess their methodological as well as reporting quality, respectively. Furthermore, we analyzed the confounding factors by indirect comparisons based on the meta-analyses.

### Quality assessment

Methodological and reporting quality of meta-analysis and SRs are crucial to public health and clinical decision-making. In the current study, we used the AMSTAR-2 checklist to assess the methodological quality. The methodological quality of the SRs was classified as critically low, and the adherence rate of individual items was suboptimal. Research protocols help to increase the transparency of the review methods and avoid bias in outcome reporting. The previous study has shown that prospective registration could effectively improve the overall methodological and reporting quality of systematic reviews. However, none of the SRs provided an explicit statement that the review methods were established before the conduct of the review and clarified the significant deviations from the protocol and reported on the sources of funding for the studies included in the review. 80.6% of SRs did not account for RoB in primary studies when interpreting/discussing the results of the review. 80.6% SRs did not explain the selection of the study design for inclusion and provide a list of exclusion studies and justify the exclusions PRISMA results showed that 9.6% of the included studies had serious, 35.4% had minor, and 54.8% had minimal reporting flaws. The scientific quality of individual studies affected the findings of this meta-analysis [54], although some of the articles did not report methods for assessing the risk of bias of different studies. For example, Cohen et al. [54] suggested searching Chinese databases may yield substantial additional clinical evidence, and recommended by the Chinese Biomedical Database for retrieval of SR literature. However, most SRs and meta-analyses are limited to English. In addition, some items did not have good performance, such as a structured summary, risk of bias, search, results of individual studies, and especially the protocol registration. Currently, the PRISMA checklist provides excellent guidelines for developing a high-quality SR [55], although improvement of the reporting quality is still needed.

### Relationship between the risk of urinary cancer and obesity, overweight, and BMI

Our comprehensive analysis revealed a strong association between kidney cancer and obesity, overweight, and BMI increase. On the other hand, a mild relationship was observed between the incidence of prostate and bladder cancers with obesity, overweight, and BMI increase, in line with Renehan et al.’s work [43].

### Subgroup analysis

We detected heterogeneity among the SRs and meta-analyses included in this study. To further demonstrate the relationships between the risk of urinary cancer and obesity, we performed subgroup analysis targeting major potential confounders such as gender, age, methods of assessing BMI, geographic location, physical activity, and family history of cancer. The results revealed a stronger association between the incidence of kidney and prostate cancers and BMI increase (5 kg/m^2^) in Asian populations compared to European and North American groups. This association was also evident between overweight and kidney cancer, although this was different from previous studies [39, 43]. The association between the incidence of kidney cancer and obesity, overweight, and BMI increase (5 kg/m^2^) was more influential in women than in men. However, we did not find convincing evidence to support the existence of differences across genders with regard to the relationship between BMI increase and incidence of bladder cancer. Age (> 50 years) was a confounding factor in the relationship between obesity and the prevalence of bladder cancer, although this trend was reversed in people with kidney cancer. Additionally, alcohol consumption was a confounding risk factor in the association between obesity and the incidence of bladder cancer.

### Mortality of different urinary cancers

Our results revealed a mild association between mortality rate in prostate and bladder cancers with increased BMI (5 kg/m^2^), whereas kidney cancer had a strong relationship, consistent with previous reports [39]. Increased BMI (5 kg/m^2^) also showed a strong association with mortality rates from prostate cancer, although this impact was not significant in all-cause mortality of prostate cancer. Although previous reports have confirmed that small sample size studies do not affect the results in the absence of obvious methodological deficiencies and marked differences among studies, it is still possible that a small sample size may affect our assessment of the association between increased BMI and urinary cancers [56].

### Limitations

Our study had several limitations. Firstly, our search criteria were limited to SRs and meta-analyses written in English, which may lead to language bias. Some studies have reported good-quality SRs published using other languages [57, 58]. Secondly, our findings including assessments of certainty of evidence are based on the information provided by the authors of the reviews, and we have not retrieved or evaluated data from any primary studies. Thirdly, we comprehensively performed subgroup analyses; however, it was unclear how original systematic reviews were classified for variables such as BMI self-reported and physical activity [39], and we did not perform all subgroup analyses to evaluate the impact of other factors on the incidence of three kinds of urinary cancers as prior defined due to limited data. Fourth, while it could be expected that there would be some overlap of primary articles within included SRs, we have not systematically explored these overlaps. Consequently, this may lead to inaccuracies in the reporting of data such as the numbers of participants and primary studies and may contribute to “double counting” of data within reported meta-analyses. Fifth, due to limited data, we did not perform subgroup analyses to evaluate the impact of BMI increase on mortality of urinary cancers.

## Conclusions

In summary, our results provide new epidemiological evidence to affirm the association between incidence and mortality of three types of urinary cancer with overweight and obesity. To minimize the impact of the diseases, the public should be informed about the benefits of weight management. The findings on differences across age, geographic location, genders, and alcohol consumption further provide valuable evidence that can guide the prevention of urinary cancers.

## Supplementary Information

**Additional file 1.** PRISMA 2009 Checklist.

**Additional file 2.** Search Strategy for PubMed.

## Data Availability

All data generated or analyzed during this study are included in this published article [and its supplementary information files].

## Abbreviations

SR: Systematic review
RR: Relative risk
OR: Odds ratio
HR: Hazard ratio
CI: Confidence interval
BMI: Body mass index
AMSTAR-2: Assessment of Assessment of Multiple Systematic Reviews-2
PRISMA: Preferred Reporting Items for Systematic Reviews and Meta-analyses
