# Supplementary material for: Associating the risk of three urinary cancers with obesity and overweight: an overview with evidence mapping of systematic reviews
Source: Syst Rev. 2021 Feb 17;10:58. doi: 10.1186/s13643-021-01606-8 (PMC7888186; doi:10.1186/s13643-021-01606-8)
Supplement: Supplementary file 2 — Additional file 2. Search Strategy for PubMed. [file 13643_2021_1606_MOESM2_ESM.pdf]

Searching strategy of Pubmed

#1 "Meta-Analysis" [Publication Type] OR "Network Meta-Analysis"[Mesh] OR "Meta-Analysis as Topic"[Mesh]

#2 "Meta-Analysis"[Title/Abstract] OR "Meta Analysis"[Title/Abstract] OR "Meta-Analyses"[Title/Abstract] OR "Meta Analyses"[Title/Abstract] OR "gathering analysis"[Title/Abstract] OR "Meta Analysis"[Title/Abstract] OR "Systematic evaluation"[Title/Abstract] OR "Systematic assessment"[Title/Abstract] OR "Systematic review"[Title/Abstract] OR "Systematic reviews"[Title/Abstract] OR "System evaluation"[Title/Abstract] OR "System Assessment"[Title/Abstract] OR "Systemic review"[Title/Abstract] OR "Systemic reviews"[Title/Abstract]

#3 "Urologic Neoplasms"[Mesh] OR "Urinary Bladder Neoplasms"[Mesh]) OR "Urogenital Neoplasms"[Mesh]

#4 "Urological Neoplasms"[Title/Abstract] OR "Urological Neoplasm"[Title/Abstract] OR "Urinary Tract Neoplasms"[Title/Abstract] OR "Urinary Tract Neoplasm"[Title/Abstract] OR "Urologic Neoplasms"[Title/Abstract] OR "Urologic Neoplasm"[Title/Abstract] OR "Cancer of Urinary Tract"[Title/Abstract] OR "Urinary Tract Cancers"[Title/Abstract] OR "Urological Cancer"[Title/Abstract] OR "Urological Cancers"[Title/Abstract] OR "Cancer of the Urinary Tract"[Title/Abstract] OR "Urinary Tract Cancer"[Title/Abstract] OR "Urinary Tract Cancers"[Title/Abstract] OR "Urologic Cancer"[Title/Abstract] OR "Urologic Cancers"[Title/Abstract] OR "Kidney Neoplasm"[Title/Abstract] OR "Renal Neoplasms"[Title/Abstract] OR "Renal Neoplasm"[Title/Abstract] OR "Cancer of Kidney"[Title/Abstract] OR "Kidney Cancers"[Title/Abstract] OR "Renal Cancer"[Title/Abstract] OR "Renal Cancers"[Title/Abstract] OR "Cancer of the Kidney"[Title/Abstract] OR "Kidney Cancer"[Title/Abstract] OR "Renal Cell Carcinomas"[Title/Abstract] OR "Renal Cell Adenocarcinoma"[Title/Abstract] OR "Renal Cell Adenocarcinomas"[Title/Abstract] OR "Adenocarcinoma Of Kidney"[Title/Abstract] OR "Adenocarcinoma Of Kidneys"[Title/Abstract] OR "Renal Cell Cancer"[Title/Abstract] OR "Renal Cell Cancers"[Title/Abstract] OR "Renal Adenocarcinoma"[Title/Abstract] OR "Renal Adenocarcinomas"[Title/Abstract] OR "Nephroid Carcinoma"[Title/Abstract] OR "Nephroid Carcinomas"[Title/Abstract] OR "Renal Cell Carcinoma"[Title/Abstract] OR "Chromophobe Renal Cell Carcinoma"[Title/Abstract] OR

"Sarcomatoid Renal Cell Carcinoma"[Title/Abstract] OR "Papillary Renal Cell Carcinoma"[Title/Abstract] OR "Renal Cell Carcinoma, Papillary"[Title/Abstract] OR "Chromophil Renal Cell Carcinoma"[Title/Abstract] OR "Clear Cell Renal Cell Carcinoma"[Title/Abstract] OR "Grawitz Tumor"[Title/Abstract] OR "Clear Cell Renal Carcinoma"[Title/Abstract] OR "Hypernephroid Carcinoma"[Title/Abstract] OR "Hypernephroid Carcinomas"[Title/Abstract] OR "Hypernephroma"[Title/Abstract] OR "Hypernephromas"[Title/Abstract] OR "Collecting Duct Carcinoma (Kidney)"[Title/Abstract] OR "Collecting Duct Carcinomas (Kidney)"[Title/Abstract] OR "Collecting Duct Carcinoma of the Kidney"[Title/Abstract] OR "Renal Collecting Duct Carcinoma"[Title/Abstract] OR "Collecting Duct Carcinoma"[Title/Abstract] OR "Collecting Duct Carcinomas"[Title/Abstract] OR "Mesoblastic Nephromas"[Title/Abstract] OR "Mesoblastic Nephroma"[Title/Abstract] OR "Congenital Mesoblastic Nephromas"[Title/Abstract] OR "Congenital Mesoblastic Nephroma"[Title/Abstract] OR "Wilms Tumor"[Title/Abstract] OR "Wilms' Tumor"[Title/Abstract] OR "Wilm Tumor"[Title/Abstract] OR "Nephroblastoma"[Title/Abstract] OR "Nephroblastomas"[Title/Abstract] OR "Urologic Neoplasms"[Title/Abstract] OR "Ureteral Neoplasm"[Title/Abstract] OR "Ureteral Neoplasms"[Title/Abstract] OR "Ureter Neoplasms"[Title/Abstract] OR "Ureter Neoplasm"[Title/Abstract] OR "Neoplasms of Ureter"[Title/Abstract] OR "Cancer of Ureter"[Title/Abstract] OR "Ureter Cancers"[Title/Abstract] OR "Ureter Cancer"[Title/Abstract] "Ureteral Cancer"[Title/Abstract] OR "Ureteral Cancers"[Title/Abstract] OR "Cancer of the Ureter"[Title/Abstract] OR "Urethral Neoplasm"[Title/Abstract] OR "Urethra Neoplasms"[Title/Abstract] OR "Urethra Neoplasm"[Title/Abstract] OR "Cancer of Urethra"[Title/Abstract] OR "Urethra Cancers"[Title/Abstract] OR "Urethra Cancer"[Title/Abstract] OR "Urethral Cancer"[Title/Abstract] OR "Urethral Cancers"[Title/Abstract] OR "Cancer of the Urethra"[Title/Abstract] OR "Urinary Bladder Neoplasm"[Title/Abstract] OR "Bladder Neoplasms"[Title/Abstract] OR "Bladder Neoplasm"[Title/Abstract] OR "Bladder Tumors"[Title/Abstract] OR "Bladder Tumor"[Title/Abstract] OR "Urinary Bladder Cancer"[Title/Abstract] OR "Malignant Tumor of Urinary Bladder"[Title/Abstract] OR "Cancer of the Bladder"[Title/Abstract] OR "Bladder Cancer"[Title/Abstract] OR "Bladder Cancers"[Title/Abstract] OR "Cancer of Bladder"[Title/Abstract]

#5 "Overweight"[Title/Abstract] OR "Obesity Hypoventilation Syndrome"[Title/Abstract] OR "Abdominal Obesity"[Title/Abstract] OR "Metabolically Benign Obesity"[Title/Abstract] OR "Morbid Obesity"[Title/Abstract] OR "Pediatric Obesity"[Title/Abstract] OR "Prader-Willi Syndrome"[Title/Abstract] OR "Short Stature-Obesity Syndrome"[Supplementary Concept] OR "Adiposity"[Mesh] OR "Abdominal Fat"[Mesh] OR "Intra-Abdominal Fat" [Title/Abstract] OR "Abdominal Subcutaneous Fat" [Title/Abstract] OR "Body Mass Index"[Title/Abstract] OR "Body Mass Index" [Title/Abstract] OR "Obesity" [Title/Abstract] OR "BMI"[Title/Abstract]

#6 "Body Mass Index"[Mesh] OR "Obesity"[Mesh] OR "Overweight"[Mesh] OR "Adiposity"[Mesh] OR "Abdominal Fat"[Mesh]

#7 #1 OR #2

#8 #3 OR #4

#9 #5 OR #6

#10 #7 AND #8 AND #9
